# Epigenetic silencing of BCL6B inactivates p53 signaling and causes human hepatocellular carcinoma cell resist to 5-FU

**DOI:** 10.18632/oncotarget.3413

**Published:** 2015-03-21

**Authors:** Xin Li, Jie Yu, Malcolm V. Brock, Qian Tao, James G. Herman, Ping Liang, Mingzhou Guo

**Affiliations:** ^1^ Department of Interventional Ultrasound, Chinese PLA General Hospital, Beijing, China; ^2^ Oncology Center, Johns Hopkins University, Baltimore, Maryland, USA; ^3^ Department of Clinical Oncology, The Chinese University of Hong Kong, Hong Kong, China; ^4^ Department of Gastroenterology & Hepatology, Chinese PLA General Hospital, Beijing, China

**Keywords:** BCL6B, DNA methylation, hepatocellular carcinoma, p53 signaling, 5-fluorouracil

## Abstract

BCL6B is a potential tumor suppressor in human gastric cancer, but the regulation and mechanism of BCL6B in human hepatocellular carcinogenesis remain unclear. This study is to explore the epigenetic change and mechanism of BCL6B in human hepatocellular carcinoma (HCC). Nineteen hepatic cancer cell lines, 50 cases of adjacent tissue and 149 cases of HCC samples were employed. BCL6B is methylated in 100% (19/19) of human HCC cell lines, 40.0% (20/50) of adjacent tissue samples and 86.6% (129/149) of primary cancer samples. Methylation of BCL6B is associated with HBV positive (*p* < 0.05). But no association was found with age, sex, tumor size, differentiation, TNM stage, recurrence and survival. Loss of BCL6B expression was found in 19 of completely methylated HCC cell lines. BCL6B was re-expressed after 5-aza-2′-deoxycytidine treatment. Restoration of BCL6B expression suppressed cell proliferation, induced apoptosis and G1/S arrest in HCC cells. The expression of EGR1, a key component of p53 signaling, was increased after re-expression BCL6B in HCC cells. Re-expression of BCL6B activated p53 signaling and sensitized HCC cells to 5-fluorouracil. BCL6B is frequently methylated in human HCC and the expression of BCL6B is regulated by promoter region hypermethylation. BCL6B activates p53 signaling by increasing EGR1 expression in HCC.

## INTRODUCTION

Hepatocellular carcinoma (HCC) is the fifth most malignant cancer and the third leading cause of cancer-related death worldwide [1, 2]. Even through improved interventional and surgical treatment, The 5-year survival rate is less than 15%. The major reason is cancer recurrence and metastasis [3–5]. Emerging evidence indicates that epigenetic alterations, particularly inactivation of tumor suppressor genes or tumor-related genes via promoter region hypermethylation, play an important role in the development and progression of HCC [6–9]. B cell CLL/lymphoma 6 member B (BCL6B) is a homologue of B cell CLL/lymphoma 6 (BCL6). It is located on chromosome 17p13.1, a region of 800 bp next to p53 gene. BCL6B was regarded as a potential tumor suppressor and methylation of BCL6B may serve as a poor prognosis marker in gastric cancer [10–12]. In this study, we analyzed the epigenetic changes and the mechanism of BCL6B in hepatocellular carcinogenesis.

## RESULTS

### BCL6B expression was regulated by promoter region hypermethylation in HCC cell lines

**Figure 1:**
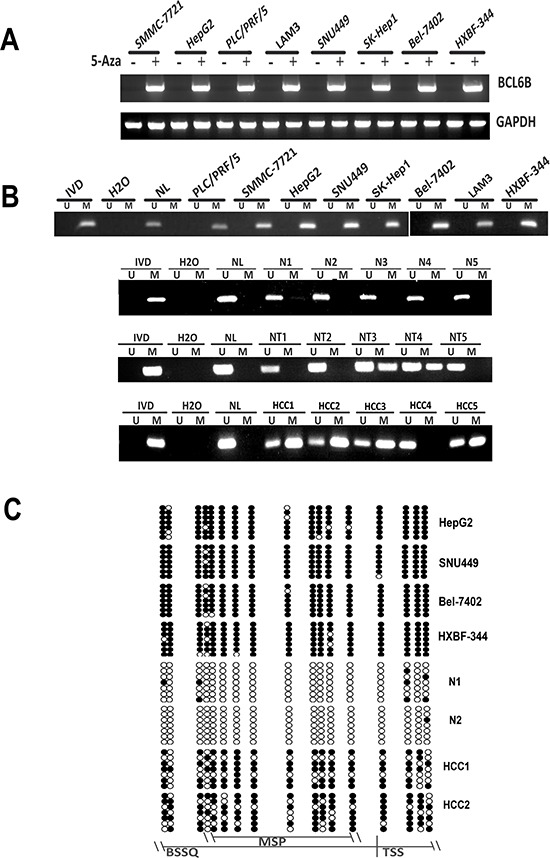
BCL6B expression was silenced by promoter region hypermethylation in HCC **A.** The expression of BCL6B was detected by semi-quantitative RT-PCR before and after 5-Aza treatment in HCC cell lines. GAPDH: internal control. (+): 5-Aza treated, (−):5-Aza untreated; SMMC-7721, HepG2, PLC/PRF5, LAM3, SNU449, Sk-hep1, Bel-7402 and HBXF344 are HCC cell lines **B.** Methylation specific PCR results of BCL6B gene in human HCC. U: unmethylated allele; M: methylated allele; IVD: *in vitro* methylated DNA; NL: normal lymphocyte DNA; NI, N2, N3, N4, N5: non-cancerous liver tissue samples: NT1, NT2, NT3, NT4 NT5: non-cancerouse tissue; HCC1, HCC2, HCC3, HCC4, HCC5: primary HCC samples. **C.** BSSQ results of BCL6B promoter region in HepG2, SNU449, HXBF344, BeL-7402, N1, N2, HCC1, HCC2. MSP: MSP amplification region; Filled circle: methylated CpG sites; opened circle: unmethylated CpG sites; TSS: transcription start site; BSSQ: BSSQ amplification region.

To explore the expression of BCL6B in HCC cells, semi-quantitative RT-PCR was employed. Loss of BCL6B expression was found in HepG2, SNU449, PLC/PRF/5, SK-Hep1, SMMC-7721, HBXF344, LAM3, SNU182, SNU387, SNU475, Hep3B, Bel-7402, QSG7701, QGY-7703, BEL-7404, BEL-7405, MHCC97H, Huh7 and Huh1 cells (Figure 1A). The promoter region methylation was detected by MSP. MSP primers were designed around transcription start site in the CpG island of BCL6B gene promoter region. Complete methylation was found in HepG2, SNU449, PLC/PRF/5, SK-Hep1, SMMC-7721, HBXF344, LAM3, SNU182, SNU387, SNU475, Hep3B, Bel-7402, QSG7701, QGY-7703, BEL-7404, BEL-7405, MHCC97H, Huh7 and Huh1 cells (Figure 1B). To further validate MSP results and the density of promoter region methylation, bisulfite sequencing was performed in HepG2, SNU449, Bel-7402 and HBXF344 cells. As shown in Figure 1C, MSP results are correlated with bisulfite sequencing results very well. HepG2, SNU449, Bel-7402 and HBXF344 cells are densely methylated. These results suggest that BCL6B expression is correlated with methylation status in HCC cells. To further validate the expression of BCL6B is regulated by promoter region hypermethylation, 5-Aza, a DNA methylation transferase inhibitor, was applied in these HCC cells. Re-expression of BCL6B was found in HepG2, SNU449, PLC/PRF/5, SK-Hep1, SMMC-7721, HBXF344, LAM3, SNU182, SNU387, SNU475, Hep3B, Bel-7402, QSG7701, QGY-7703, BEL-7404, BEL-7405, MHCC97H, Huh7 and Huh1 cells (Figure 1A). These results further suggest that BCL6B expression was regulated by promoter region methylation in HCC.

### BCL6B is frequently methylated in human primary HCC and reduced expression was associated with promoter region hypermethylation

**Figure 2:**
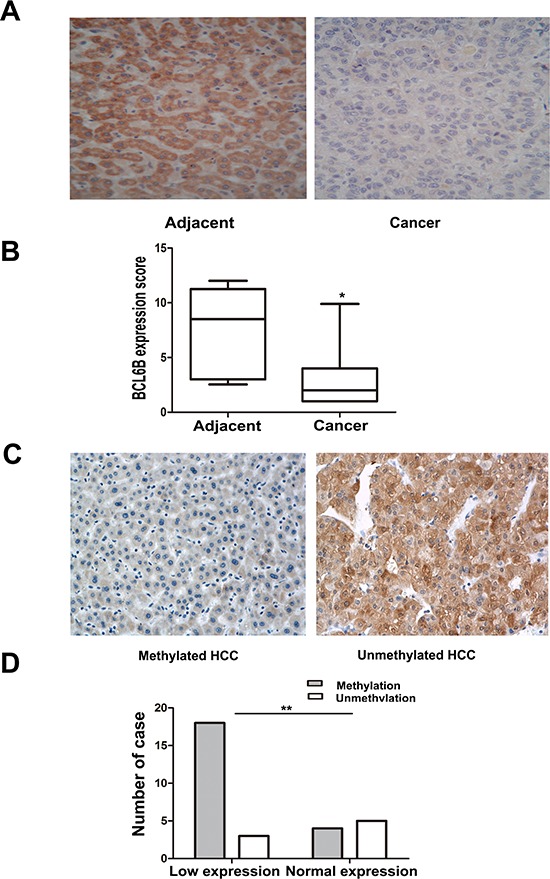
Reduced expression of BCL6B was associated with promoter region hypermethylation in human primary HCC **A.** Representative BCL6B staining in matched human primary HCC (right) and adjacent tissue samples (left) by IHC. (X200). **B.** The scores of BCL6B expression in 30 matched HCC and adjacent tissue samples are shown as box plots. Horizontal lines in the boxes represent the median score; the bottom and top lines of the boxes represent the 25th and 75th percentiles, respectively; vertical bars represent the range of data. (*p < 0.05, **p < 0.01) **C.** Representative BCL6B staining in methylated (left) and unmethylated HCC (right) by IHC. (X200) **D.** Loss of/reduced expression of BCL6B was associated with promoter region hypermethylation in human HCC. (*p < 0.05, **p < 0.01)

To investigate the methylation status of BCL6B, 149 cases of primary HCC, 50 cases of cancer adjacent tissue and 8 cases of normal liver tissue samples were examined by MSP. As shown in Figure 1B & 1C, 86.6% (129/149) of HCC and 40.0% (20/50) of adjacent tissue samples were methylated, but no methylation was found in normal liver tissue samples. Methylation of BCL6B is associated with HBV positive HCC (*p* < 0.05). No association was found between BCL6B methylation and hepatitis in adjacent tissue samples (*p* > 0.05). Existing hepatitis was evaluated by elevated the level of aspartate aminotransferase (AST) and alanine aminotransferase (ALT). No association was found between BCL6B methylation and age, gender, tumor size, cell differentiation and TNM stage (Table 1). The expression of BCL6B was evaluated by IHC in 30 cases of available HCC and matched adjacent tissue samples. Reduced expression was found in 21 cases of cancer samples and 9 cases of adjacent tissue samples (Figure 2A). Reduced expression of BCL6B is significantly in cancer tissue compared with adjacent tissue samples (Figure 2B, *p* < 0.05). In 21 cases of BCL6B reduced cancer samples, 18 cases were methylated (Figure 2C). Reduced expression was associated with promoter region hypermethylation significantly (Figure 2D, *p* < 0.01). It suggests that BCL6B is possibly regulated by promoter region methylation in human primary HCC.

**Table 1:** Univariate analysis of relationship between BCL6B methylation and clinicopathologic features of HCC patients

BCL6B methylation status					
Variable	No.	Methylated (*N* = 129)	Unmethylated (*N* = 20)	χ 2	*P* value^a^
**Gender**				3.47	0.06
Male	88	80	8		
Female	61	49	12		
**Age (years)**				0.01	0.91
≤ 65	114	99	15		
> 65	35	30	5		
**Size (cm)**				3.78	0.15
≤ 3	67	59	8		
3–5	47	43	4		
> 5	35	27	8		
**Differentiation**				0.02	0.99
High	39	34	5		
Middle	80	69	11		
Low	30	26	4		
**Type of hepatisis**				9.14	0.03*
HBV	130	114	16		
HCV	9	5	4		
HBV + HCV	1	1	0		
N	9	9	0		
**TNM**				4.56	0.10
I	90	79	11		
II	24	23	1		
III	35	27	8		

a *P* values are obtained from chi-square test.

* Statistically significant, *p* < 0.05.

### Restoration of BCL6B expression suppresses cell proliferation, induces apoptosis and G1/S arrest in HCC cells

**Figure 3:**
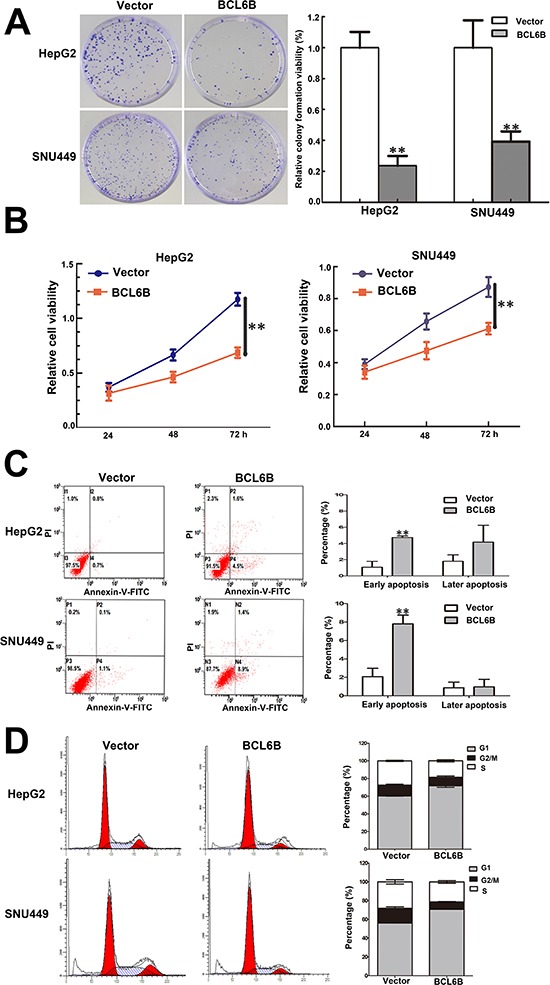
Restoration of BCL6B expression inhibited proliferation, induced aopotosis and G1/S arrest in HCC cells **A.** Colony formation in BCL6B re-expressed and unexpressed HepG2 and SNU449 cells. Each experiment was repeated for three times. **p < 0.01. **B.** Growth curves of BCL6B re-expressed and unexpressed HepG2 and SNU449 cells analyzed by CCK-8 assay. Each experiment was repeated for three times. **p < 0.01. **C.** Representative apoptosis data analyzed by flow cytometry in BCL6B re-expressed and unexpressed HepG2 and SNU449 cells. Each experiment was repeated for three times. **p < 0.01. **D.** Cell cycle distribution in BCL6B re-expressed and unexpressed HepG2 and SNU449 cells. Each experiment was repeated for three times.

The effect of BCL6B on cell proliferation was analyzed by colony formation. The colony number was 502.67 ± 50.01 vs. 118.67 ± 32.08 in HepG2 cells (*p* < 0.01) and 506.67 ± 90.89 vs. 198.67 ± 33.31 in SNU449 cells (*p* < 0.01) before and after restoration of BCL6B expression (Figure 3A). The results suggest that HCC cell colony formation was suppressed by BCL6B. The cell viability was detected by MTT. The OD value is 1.174 ± 0.058 vs. 0.687 ± 0.046 (*p* < 0.01) in HepG2 cells and 0.873 ± 0.063 vs. 0.586 ± 0.034 (*p* < 0.01) in SNU449 cells before and after restoration of BCL6B expression (Figure 3B). It indicates that cell viability was suppressed by BCL6B in HCC cells. To explore the effect of BCL6B on apoptosis, flow cytometry was employed. Early apoptosis was detected by staining phosphatidylserin (PS) with Annexin V. The ratio of apoptosis was 1.06 ± 0.72% vs. 4.73 ± 0.21% in HepG2 cells (*p* < 0.01), and 2.06 ± 0.90% vs. 7.8 ± 0.95% in SNU449 cells (*p* < 0.01) before and after re-expression of BCL6B. Late apoptosis was evaluated by propidium iodide (PI) staining for broken down DNA. The ratio of apoptosis was 1.80 ± 0.80% vs. 4.73 ± 1.75% in HepG2 cells (*p* > 0.05) and 0.86 ± 0.61% vs. 0.97 ± 0.81% in SNU449 cells (*p* > 0.05) before and after re-expression of BCL6B (Figure 3C). Above results suggest that BCL6B induces apoptosis in the early step of liver carcinogenesis.

The effect of BCL6B on cell cycle was evaluated by flow cytometry. The cell phase distribution in HepG2 cells before and after re-expression of BCL6B was as follow: G1 phase: 60.32 ± 0.31% vs. 71.73 ± 1.71% (*p* < 0.01), S phase: 27.40 ± 0.71% vs. 18.53 ± 1.39% (*p* < 0.01), G2/M phase: 12.26 ± 1.02% vs. 9.74 ± 1.39% (*p* < 0.01). The cell phase distribution in SNU449 cells before and after re-expression of BCL6B was as follow: G1 phase: 56.14 ± 1.03% vs. 70.90 ± 0.92% (*p* < 0.01), S phase: 28.10 ± 1.32% vs. 21.36 ± 1.38% (*p* < 0.05), G2/M phase: 15.77 ± 1.60% vs. 7.74 ± 0.48% (*p* < 0.01). These results indicate that G1/S arrest was induced by BCL6B (Figure 3D) in HCC cells.

### EGR1 was up-regulated by BCL6B in HCC cells

**Figure 4:**
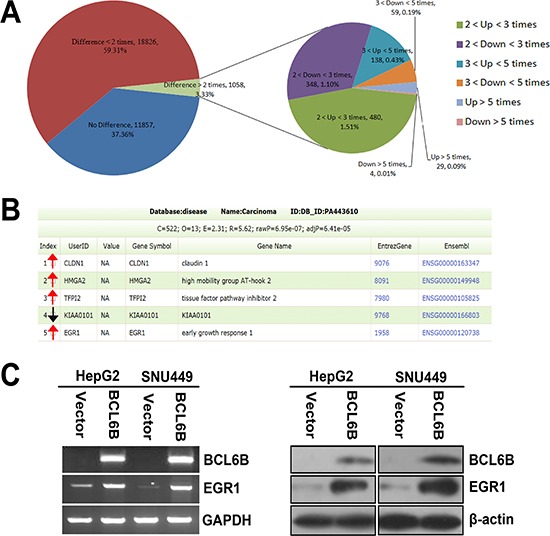
Differentially expressed genes in BCL6B re-expressed and unexpressed SNU449 cells analyzed by gene expression array **A.** The pie chart analysis for all 31741 probes in BCL6B re-expressed and unexpressed SNU449 cells. 167 genes were up regulated and 63 genes were down regulated for more than 3 times. **B.** HMGA2, CLDN1, TFPI2, KIAA0101 and EGR1 are cancer related genes according to Diseases Association Analysis. HMGA2, CLDN1, TFPI2 and EGR1 which are up-regulated for 5, 3.3, 3.7 and 31.4 times, and KIAA0101 is down-regulated for 3.2 times, respectively. (Red arrow stands for up-regulated and black arrow stands for down-regulated genes) **C.** The expression of EGR1 was validated by semi-quantitative RT-PCR and Western Blot in BCL6B unexpressed and re-expressed HepG2 and SNU449 cells.

To understand the mechanism of BCL6B on HCC carcinogenesis, gene expression microarray was employed in this study. As shown in Figure 4A, 167 genes were up-regulated and 63 genes were down-regulated over 3 times after re-expression of BCL6B in SNU449 cells. Among these genes, HMGA2, CLDN1, TFPI2, KIAA0101 and EGR1 are cancer related genes according to Diseases Association Analysis (http://bioinfo.vanderbilt.edu/webgestalt/). HMGA2, CLDN1, TFPI2 and EGR1 are up-regulated for 5, 3.3, 4.8 and 31.4 times, and KIAA0101 was down-regulated for 3.2 respectively (Figure 4B). Even through, the tendency of expression changes of these genes, which detected by quantitative RT-PCR, was similar with microarray analysis (Supplementary Figure S1). Apparent increasing expression was only found in EGR1 gene by semi-quantitative RT-PCR and western blot in BCL6B re-expressed HepG2 and SNU449 cells (Figure 4C).

### Restoration of BCL6B expression activates p53 signaling

**Figure 5:**
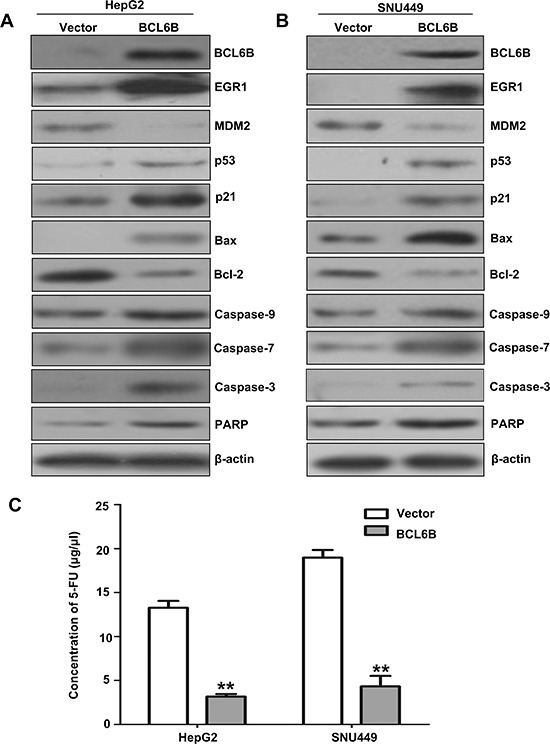
The effect of BCL6B on p53 signaling and apoptosis in HCC cells **A&B.** The expression of BCL6B, EGR1, MDM2, p53, p21, Bax, bcl-2, caspase-9, caspase-7, caspase-3 and PARP was detected by western blot in BCL6B unexpressed and re-expressed HepG2 and SNU449 cells. β-actin: internal control. **C.** IC50 value of 5-FU in BCL6B unexpressed and re-expressed HepG2 and SNU449 cells. The IC50 value of 5-FU was evaluated by CCK-8 activity. White columns: the IC50 value of 5-FU in BCL6B unexpressed HepG2 and SNU449 cells. Gray columns: the IC50 value of 5-FU in BCL6B re-expressed HepG2 and SNU449 cells.

EGR1 is a component of p53 signaling, which suppresses cell growth in human lung cancer [13–17]. To analyze the effect of BCL6B in p53 signaling in HepG2 and SNU449 cells, first we eliminated the possible effect of p53 mutation on these cells. No p53 mutation was found in HepG2 cells. While SNU449 carries a G to A substitution at nucleotide 481 of the p53 gene, leading to an alanine to threonine mutation at codon 161 [18]. But this mutation site is not related to the interacting region of p53 with EGR1 [19]. To further understand the role of BCL6B in HCC, the expression of EGR1 and p53 were detected before and after re-expression of BCL6B in HepG2 and SNU449 cells. The expression of EGR1 and p53 were increased apparently after restoration of BCL6B expression in HepG2 and SNU449 cells. As shown in Figure 5A & 5B, the expression of p21, caspase-3, caspase-7, caspase-9, PARP and Bax were increased and the expression of MDM2 and bcl-2 were reduced in BCL6B expressed HepG2 and SNU449 cells. These results suggest that p53 signaling was activated by BCL6B through EGR1. And BCL6B induce apoptosis in HCC cells.

### P53 signaling was inhibited by knocking down BCL6B or EGR1 in DKO cells

**Figure 6:**
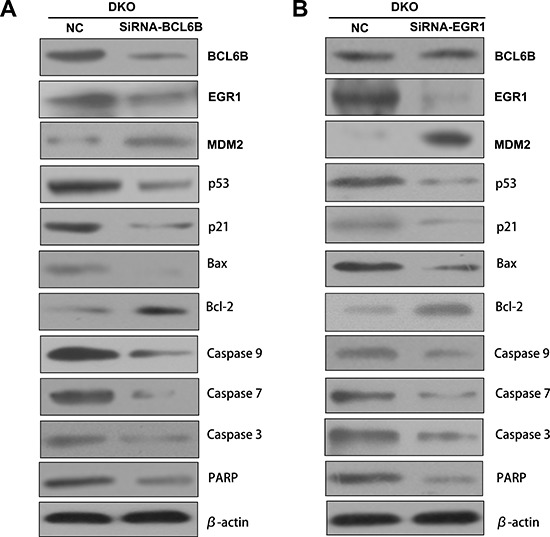
P53 signaling was activated by BCL6B through EGR1 in DKO cell line **A.** The expression of BCL6B, EGR1, MDM2, p53, p21, Bax, bcl-2, caspase-9, caspase-7, caspase-3 and PARP was detected by western blot in BCL6B expressed and siRNA knocking down DKO cell line. β-actin: internal control. **B.** The expression of BCL6B, EGR1, MDM2, p53, p21, Bax, bcl-2, caspase-9, caspase-7, caspase-3 and PARP was detected by western blot in EGR1 expressed and siRNA knocking down DKO cell line. β-actin: internal control.

To validate p53 signaling was activated by BCL6B via EGR1, BCL6B and EGR1expressed DKO cells was employed. As shown in Figure 6, the expression of EGR1, p53, p21, Bax, caspase-3, caspase-7, caspase-9 and PARP were reduced and MDM2 and bcl-2 were increased by knocking down BCL6B (Figure 6A). Similar to BCL6B knocking down, the expression of p53, p21, Bax, caspase-3, caspase-7, caspase-9 and PARP were reduced and MDM2 and bcl-2 were increased after knock down EGR1, while no change was found for BCL6B expression (Figure 6B). These results indicate p53 signaling was activated by BCL6B via EGR1.

### Restoration of BCL6B expression sensitized HCC cells to 5-FU

The tumor suppressor p53 maintains DNA integrity by transcriptionally activating genes such as p21 (CDKN1N) and GADD45a, the products of which induce cell-cycle arrest in response to DNA damage [20–22]. However, depending on the cellular context and the nature of the DNA damage, p53 can trigger elimination of the damage cells by promoting apoptosis through the induction of pro-apoptosis genes, such as Bax, and the down-regulation of anti-apoptosis Bcl2 [23, 24]. *In vitro* studies have reported that loss of p53 function reduces cellular sensitivity to 5-FU [25, 26]. Disrupting both alleles of TP53 or Bax in colon cancer cell line made the cells strikingly resistant to apoptosis induced by 5-FU compared with the parental line [25, 27, 28]. 5-FU is widely used for cancer treatment and still the mainstay of chemotherapy. Apoptosis was induced by activating p53 signaling after restoration of BCL6B expression in HepG2 and SNU449 cells. To see if BCL6B is involved in 5-FU sensitivity in HCC cells, the sensitivity of HepG2 and SNU449 cells to 5-FU was analyzed before and after re-expression of BCL6B. The IC50 of 5-FU was 13.27 ± 0.77 vs. 3.18 ± 0.28 μg/ml (*p* < 0.01) in HepG2 and 18.98 ± 0.86 versus 4.33 ± 1.18 μg/ml (*p* < 0.01) in SNU449 cells before and after restoration of BCL6B expression (Figure 5C). The results suggest that BCL6B sensitized HCC cells to 5-FU.

## DISCUSSION

BCL6B is frequently methylated in human hepatocellular carcinoma and the expression of BCL6B was regulated by promoter region hypermethylation. It suggests that BCL6B is a potential HCC detection marker and BCL6B methylation may involved in hepatocellular carcinogenesis. Increasing evidence reveals that viral genes are one of the key players in regulating DNA methylation. Particularly in cancers closely associated with the hepatitis B virus, simian virus 40 (SV40), and Epstein-Barr virus [29]. *In vitro*, HBx has been documented to disturb the host DNA methylation system by up-regulating DNMT expression and activities, which in turn results in hypermethylation and repression of some tumor suppressor genes (insulin-like growth factor binding protein-3 gene, E-cadherin), thus increasing tumor susceptibility [29–31]. Previous studies have shown that HCC is associated with hypermethylation of several tumor suppressor genes [32, 33]. We and other groups have shown that some genes are preferentially methylated in HCV-related HCC [6, 34]. In this study, we found that BCL6B methylation is related to HBV positive HCC. The result further suggests that HBV may promote hepatocellular carcinogenesis by inducing promoter region methylation in tumor suppressor. Further study found that BCL6B suppresses cell proliferation and induces apoptosis and G1/S arrest in HCC cells. To further understand the mechanism of BCL6B on hepatocellular carcinogenesis, gene expression microarray was employed. By analyzing the expression changes of genes before and after re-expression of BCL6B in methylated HCC cell lines, 167 genes were up-regulated and 63 genes were down-regulated over 3 times after re-expression of BCL6B in SNU449 cells. Among these genes, HMGA2, CLDN1, TFPI2, KIAA0101 and EGR1 are cancer related genes according to Diseases Association Analysis and increased expression of EGR1 was confirmed by further semi-quantitative RT-PCR, quantitative RT-PCR and western blot in BCL6B re-expressed HepG2 and SNU449 cells. EGR1 was reported to suppress cell growth by regulating p53 in lung cancer cells and BCL6B was reported involved in p53 signaling in gastric cancer [11, 15–17]. Thus, we focused on the p53 signaling pathway in human HCC. The expression of EGR1, p53, p21, caspase-3, caspase-7, caspase-9, PARP and Bax was increased and the expression of MDM2 and bcl-2 was reduced after restoration of BCL6B expression in human HCC cells. These results suggest that BCL6B activated p53 signaling and induced apoptosis in human HCC cells. As p53 expression was regulated by EGR1 in human lung cancer cells [15, 17]. It is reasonable to suggest that p53 signaling was activated by BCL6B through EGR1 in human HCC. It was reported before that p53 disruption rendered cells strikingly resistant to the effects of the antimetabolite 5-FU in colorectal cancer [25]. We further analyzed the sensitivity of BCL6B re-expressed and unexpressed HCC cells to 5-FU. The results demonstrated that BCL6B sensitized HCC cells to 5-FU. These results indicate that BCL6B sensitizes HCC cell to 5-FU by activating p53 signaling.

In conclusion, BCL6B is frequently methylated in human HCC and the expression of BCL6B was regulated by promoter region hypermethylation. BCL6B suppresses cell proliferation and induces apoptosis in HCC cells. EGR1, a key component of p53 signaling, was up regulated by BCL6B in HCC cells. BCL6B activates p53 signaling and sensitizes HCC cells to 5-FU.

## MATERIALS AND METHODS

### Primary human hepatic cancer samples and cell lines

One hundred and forty-nine cases of primary hepatic cancer were collected as fresh frozen tissue from Chinese PLA General Hospital. All cases of hepatic cancer were classified by TNM stage, including stage I (*N* = 90), stage II (*N* = 24) and stage III (*N* = 35). Eight cases of normal live tissue were collected as fresh frozen tissue from hepatic hemangioma patients and 50 cases of adjacent tissue samples were from primary hepatic cancer patients in Chinese PLA General Hospital. Among 149 cancer samples, 30 cases of paraffin blocks are available with matched adjacent tissue. All samples were collected under the approved guidelines of the Chinese PLA General Hospital's institutional review board.

Nineteen human hepatic cancer cell lines HepG2, SNU449, PLC/PRF/5, SK-Hep1, SMMC-7721, HBXF344, LAM3, SNU182, SNU387, SNU475, QSG7701, QGY-7703, BEL-7404, BEL-7405, MHCC97H, Huh1, Huh7, Hep3B and Bel-7402, and DKO cells (double knockouting of DNMT1 and DNMT3b in HCT116 cell) were included in this study [35]. All of HCC cell lines were previously established from primary hepatocellular carcinoma. Each of the cell lines were maintained in 90% RPMI 1640 (Intrivogen, Carlsbad, CA, USA), supplemented with 10% fetal bovine serum (Hyclone, Logan, UT). Cells were passaged 1:3 once total confluence (approximately 10^6^ cells) was reached on a 75 cm^2^ culture flask (NEST Biotechnology, Jiangsu, China).

### 5-aza-2′-deoxycytidine (5-Aza) treatment

HCC cell lines were split to low density (30% confluence) 12 hours before treatment. Cells were treated with 5-aza-2′-deoxy -cytidine (5-Aza) (Sigma, St. Louis, MO, USA) at a concentration of 2 μM. Growth medium, conditioned with DAC at 2 uM, was exchanged every 24 h for total 96 h treatment.

### RNA isolation and semi-quantitative reverse transcription PCR

Total RNA was isolated using Trizol reagent (Life Technologies, Gaithersburg, MD) and chloroform, isopropanol precipitation. Agarose gel (1%) electrophoresis and spectrophotometric analysis (A260:280 nm ratio) was used to evaluate RNA quality and quantity. Reverse transcription of RNA (5 μg) was carried out according to the instruction of Superscript III-reverse transcriptase kit (Invitrogen, Carlsbad, CA). Following first strand synthesis, the cDNA was diluted to 100 μl using water. Subsequently, 2.5 μl of the diluted cDNA mixture was used for PCR amplification in a final volume of 25 μl. A total of 35 cycles was amplified. GAPDH was amplified with 25 cycles to ensure cDNA quality and quantity for each RT-PCR. RT-PCR primers of BCL6B are as follow: Sense: 5′-AAGCCGTATAAGTGTGAGACG-3′, antisense: 5′-CCGAGAATGTGGTAGTGCAC-3′. Amplified products were analyzed on 2% agarose gels.

### DNA extraction and bisulfite modification

HCC cells and tissue samples were digested with proteinase K. Then genomic DNA was isolated by phenol-chloroform extraction. The extracted DNA was diluted in TE buffer and stored at −20°C. 2 μg of genomic DNA was diluted in 50 μl of water. The bisulfite treatment was carried out for 16 h at 50°C. DNA samples were then purified with the Wizard DNA Clean-Up System (Promega, WI, USA), desulfonated with NaOH, and then precipitated with ethanol and resuspended in 20 μl of water.

### Methylation-specific PCR (MSP) and bisulfite sequencing (BSSQ)

MSP and BSSQ were performed as described previously [36, 37]. Each PCR assay included a methylation control, an unmethylation control and water control. MSP and BSSQ primers were designed around the transcriptional start site in the promoter region of BCL6B (NM_181844.3). The primers sequences are as follow: unmethylation-forward: 5′-TTTTGTTTTGGATTTGTTATTTGGAGAGT-3′, unmethylation-reverse: 5′-CTTAACCTCAACTCCTTTATCTAACCA-3′, methylation-forward: 5′-CGTTTTGGATTCGTTATTTGGAGAGC-3′, methylation-reverse: 5′-TAACCTCGACTCCTTTATCTAACCG-3′. BSSQ-forward: 5′-GTTTTTGAGGTTTYGTTTTYGAG-3′ BSSQ-reverse: 5′-CCTCAATCTCTTATTCTTACCC-3′. The spanning of MSP and BSSQ amplification region of BCL6B are 119bp and 227bp, respectively.

### Immunohistochemistry

Immunohistochemical staining for BCL6B was performed on 4 μm thick serial sections derived from 30 cases available matched cancer and adjacent tissue paraffin blocks using antibody against BCL6B (1:100 dilution, Abcam, Cambridge, Massachusetts, USA) as described previously [38, 39]. The staining intensity and area extent were graded according to the German semi-quantitative scoring system, which has been widely accepted and used in previous studies [40–42]. In brief, staining intensity of the nucleus, cytoplasm, and/or membrane (no staining = 0; weak staining = 1; moderate staining = 2; strong staining = 3); extent of stained cells (0% = 0, 1–24% = 1, 25–49% = 2, 50–74% = 3, 75–100% = 4). The final immunoreactive score (0 to 12) was determined by multiplying intensity scores with the extent of positivity scores of stained cells. The cutoff value is 4.

### Colony formation and cell viability assay

Cells were plated in 6-well culture plates 24 h before transfection and then were transfected with empty vector pcDNA3.1 or the pcDNA3.1-HA-BCL6B vector. The transfected cells were diluted and reseeded 24 h later. Cells were seeded at 1000 cells and 1, 500 cells per well in six-well culture plates in triplicate for HepG2 and SNU449 cells. Transfected cells were selected in 400 ug/mL G418 (Invitrogen). After 14 days, colonies were fixed with 75% ethanol for 30min and stained with 0.2% crystal violet for visualization and counting. The number of colonies was counted under the microscope.

Cells (5 × 10^3^) were seeded in 96 well plates, incubated for 24 hours and transfected with pcDNA3.1-HA-BCL6B or the empty vector pcDNA3.1. Cell viability was determined by the 3-(4, 5-dimethylthiazol-2-yl) -5-(3-carboxymethoxyphenyl)-2-(4-sulfophenyl)-2 H-tetrazolium (MTS) assay (Promega, Madison, Wisconsin, USA) at 24 h, 48 h and 72 h after treatment, respectively. Absorbance was measured on a microplate reader (Thermo Multiskan MK3 USA) at a wavelength of 490 nm. The results were plotted as means ± SD. All assays were performed in triplicate and repeated for three times. The percentage of viable cells (%) = [A_450–630_(treated) - A_450–630_(blank)]/[A_450–630_(control) - A_450–630_(blank)] × 100%. IC_50_ was defined as the concentration, which was required for 50% inhibition of cell growth. The values were calculated by nonlinear regression analysis using SPSS 17.0 software (SPSS Inc., Chicago, IL).

### Flow cytometry analysis

For cell cycle analysis, briefly, after 12 hours of synchronization by serum starvation, the HepG2 and SNU449 cells transfected with BCL6B expression vector or control empty vector 24 hours before were re-stimulated with 10% fetal bovine serum (FBS) for 24 hours. Cells were fixed in 70% ethanol and stained with 50 μg/ml propidium iodide (BD Pharmingen, San Jose, CA). The cells were then sorted by FACS Calibur (BD Biosciences, Franklin Lakes, NJ) and cell-cycle profiles were analyzed by ModFit version 2.0 (Verity Software House, Topsham, ME) as described in the previous article [7, 39]. Histograms were analyzed for cell cycle compartments using Early and late apoptosis was detected by Annexin V- FITC/propidium iodide (PI) Apoptosis Detection Kit (KeyGen Biotechnology, China).

### *In vitro* chemosensitivity testing for HCC cells

Cells were seeded in 96-well plates and treated with 5-FU at the dose of 0, 0.36, 0.73, 1.56, 3.12, 6.25, 12.5, 25, 50, 100 μg/μl. The percentage of viable cells was evaluated as described above. IC50 was defined as the concentration, which was required for 50% inhibition of cell growth.

### Gene expression microarray analysis

RNA from BCL6B expressed and unexpressed SNU449 cells were converted to cDNA labeled with fluorescent dye (Cy3). The labeled cDNA were mixed and hybridized to human gene expression array and then washed, scanned, and analyzed. The gene expression array (Agilent Human (4 × 44 k)) was provided by Agilent Technologies. The ratio represents gene expression alterations between BCL6B expressed and unexpressed groups. The expression changed more than 3-fold was further analyzed by the software of Diseases Association Analysis (http://bioinfo.vanderbilt.edu/webgestalt/) and validated by semi-quantitative RT-PCR and western blot.

### SiRNA knock down

Selected siRNAs targeting BCL6B and EGR1 and RNAi negative control duplex were used in this study. The siRNA-BCL6B sequences are as follows: siRNA duplex (sense: 5′-GACGAAGACAAACCCUAUATT-3′; antisense: 5′-UAUAGGGUUUGUCUUCGUCTT-3′); The siRNA-EGR1 sequences are as follows: siRNA duplex (sense: 5′-GCAAGAGGCAUACCAAGAUTT-3′; antisense: 5′-AUCUUGGUAUGCCUCUUGCTT-3′); RNAi negative control duplex (sense: 5′-UUCUCCGAACGUGUCACGUT T-3′; antisense: 5′-ACGUGACACGUUCGGAGAAT T-3′). RNAi oligonucleotide or RNAi negative control duplex (GenePharma Co.) was transfected into DKO cells according to the manufacturer's instructions.

### Quantitative RT-PCR

Quantitative RT-PCR was performed in triplicate with an Applied Biosystems Stepone Fast Sequence Detection System using TaqMan universal PCR master mix according to the manufacturer's protocol (Applied Biosystems). Levels of RNA expression were determined using the Stepone System software version 1.3.1 (Applied Biosystems).

### Protein preparation and western blot

Protein preparation and western blot were performed according to our previous report [43]. The following primary antibodies were used: anti-BCL6B (Abcam, Cambridge, Massachusetts, USA), anti-EGR1 (Epitomics, MA, USA), anti-MDM2 (Abgent, San Diego, U.S.), anti-p53 (Bioworld Tech, MN, USA), anti-bax (Beyotime Biotech, China), anti-bcl-2 (Cell Signalinging Technology, USA), anti-caspase 3 (Abcam, MA, USA), anti-caspase 7 (Abcam, MA, USA), anti-caspase 9 (Abcam, MA, USA) and cleaved PARP (Cell Signalinging Technology, USA), and β-actin (Beyotime Biotech, China). β-actin was used in a reprobing as a loading control. The blots were visualized using enhanced chemiluminescence (Pierce Bioscience, IL, USA).

### Statistical analysis

Statistical analysis was performed using SPSS 17.0 software (SPSS Inc., Chicago, USA). Chi-square or Fischer's exact tests were used for evaluating the relationship between methylation status and clinic-pathological characteristics. All experiment data are presented as means ± standard deviation (SD) of at least three independent experiments. Two-sided tests were used to determine significance, and *p* < 0.05 was considered statistically significant.

## SUPPLEMENTARY FIGURE AND TABLE


