# Supplementary material for: Epigenetic silencing of BCL6B inactivates p53 signaling and causes human hepatocellular carcinoma cell resist to 5-FU
Source: Oncotarget. 2015 Mar 21;6(13):11547–60. doi: 10.18632/oncotarget.3413 (PMC4484475; doi:10.18632/oncotarget.3413)
Supplement: Supplementary file 1 [file oncotarget-06-11547-s001.pdf]

SUPPLEMENTARY FIGURE AND TABLE

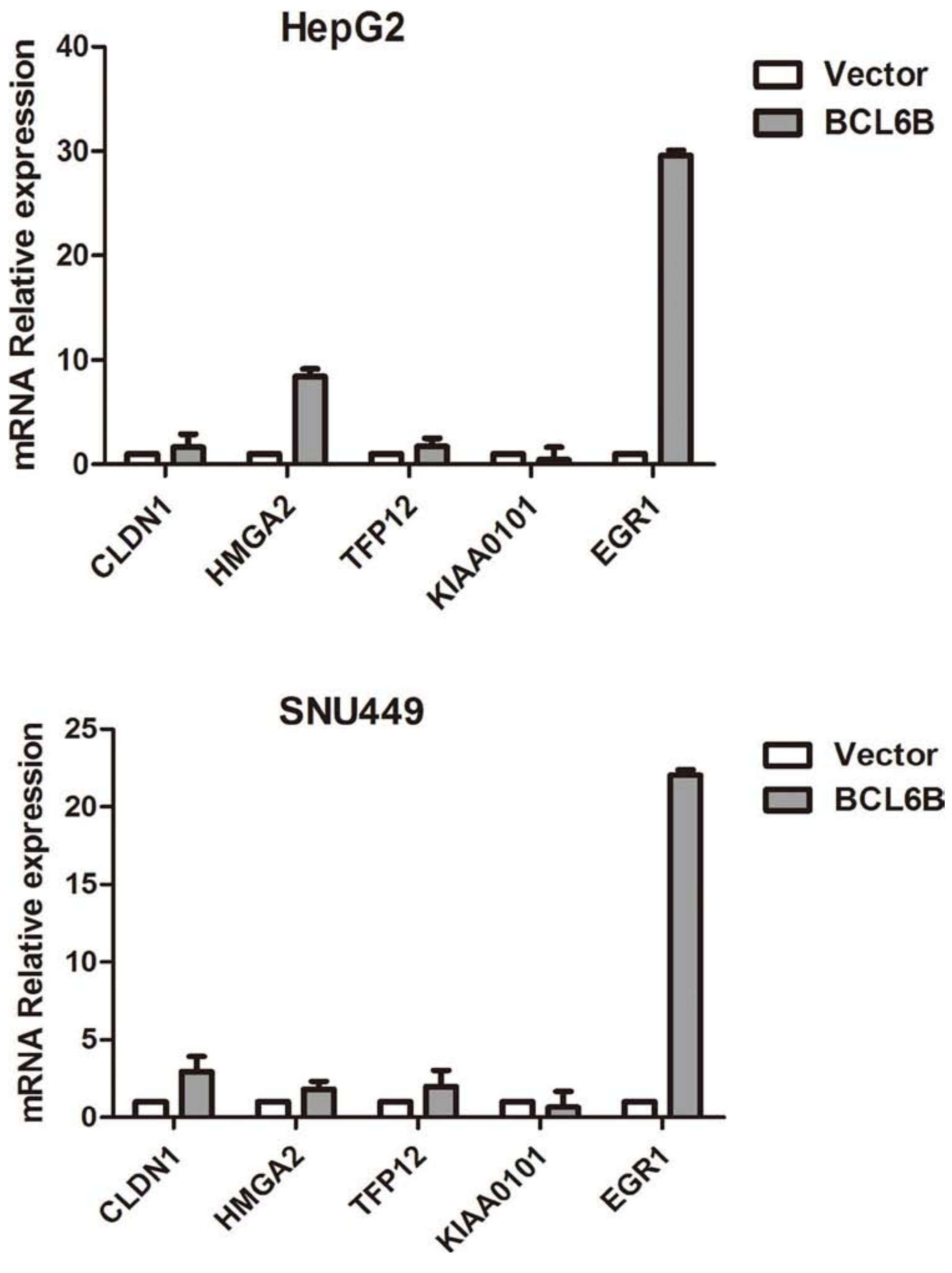

Supplementary Figure S1: The level of HMGA2, CLDN1, TFPI2, KIAA0101 and EGR1 expressed in HCC cells detected by quantitative RT-PCR before and after restoration of BCL6B expression.

Supplementary Table S1. The genes were upregulated and downregulated by BCL6B in HCC cell line

Upregulated genes (*n* = 167)		Downregulated genes (*n* = 63)	
**Gene_symbol**	**Times**	**Gene_symbol**	**Times**
EGR1	31.3815045	ATP5G3	7.111045
DHRS2	16.420902	ST6GAL1	5.310881
HAS2	9.76672	RAB26	5.165062
NA	8.991794	COQ10A	5.134877
ZACN	8.823521	CDKN2C	4.554174
AMIGO2	7.298769	IDH2	4.487811
TM6SF2	6.695016	IL17RB	4.412037
ANGPTL4	6.691899	PAFAH1B	4.260382
FEM1B	6.409032	HADH	4.145542
HTR1B	6.211841	PSMB10	3.997763
IGFBP3	6.112328	TP53INP2	3.944969
OR14J1	6.052965	MMAB	3.888405
FOSL1	6.051892	CLN8	3.863185
CTGF	5.987761	Previous version conserved probe	3.858666
ARRDC3	5.68039	Previous version conserved probe	3.810497
AMBP	5.621531	MAMLD1	3.754039
BACH1	5.570035	LHPP	3.734356
ZNF121	5.528381	NA	3.692944
IL7R	5.322968	Previous version conserved probe	3.627056
REST	5.290353	TMEM37	3.61257
TNFRSF10D	5.284688	Previous version conserved probe	3.609654
EID3	5.252144	SLC25A29	3.601757
FLJ22184	5.244801	TLR3	3.569876
IGFBP3	5.173802	CYBRD1	3.568724
SERPINE2	5.166981	NAT14	3.521993
RND3	5.098544	PPP1R3E	3.497577
HMGA2	5.065342	LDOC1	3.482483
TIPARP	5.009214	SLC22A18	3.47672
CA9	4.995435	NRM	3.427386
RELB	4.937145	SH3TC1	3.357971

(*Continued*)

Upregulated genes (*n* = 167)		Downregulated genes (*n* = 63)	
**Gene_symbol**	**Times**	**Gene_symbol**	**Times**
RORC	4.834999	DISP1	3.57857
MT1E	4.823756	PCK2	3.329287
GNAO1	4.813042	MTSS1	3.315519
TFPI2	4.811958	CPZ	3.288069
Previous version conserved probe	4.79754	SERPINI1	3.236084
ABCB9	4.795115	CD83	3.228543
HIST1H2BG	4.763129	LPIN1	3.22805
KRTAP22–1	4.74737	KIAA0101	3.221384
ADM	4.734164	ABCC3	3.214273
GAN	4.725632	TMEM14A	3.211781
KLF10	4.671177	LOC728537	3.195926
KRTAP4–5	4.657278	ZNF428	3.177968
KLHL15	4.637347	NTF3	3.173225
LAIR2	4.61979	CD70	3.17265
CYR61	4.615643	SSR1	3.169561
GADD45A	4.590169	AKR1B10	3.158617
TMEM211	4.554227	SLC25A35	3.127822
BACH1	4.531061	HAGHL	3.118208
ACTA1	4.516804	FRAT1	3.114968
JUN	4.513631	ACCS	3.112772
GABPB1	4.497107	CLN8	3.110792
ZBTB21	4.470331	LOC100507410	3.099151
NA	4.465924	Previous version conserved probe	3.096608
IER3	4.456024	Previous version conserved probe	3.096127
SENP5	4.44033	CTDSPL	3.095438
OR2F2	4.4067	CNFN	3.067764
RLIM	4.309678	LGSN	3.064031
UGCG	4.303486	Previous version conserved probe	3.06187
HAS3	4.299462	SMAD6	3.044478
RIT1	4.297867	VASH1	3.03962
SLC28A3	4.279701	POLH	2.984255

(*Continued*)

Upregulated genes (*n* = 167)		Downregulated genes (*n* = 63)	
**Gene_symbol**	**Times**	**Gene_symbol**	**Times**
TNFAIP3	4.256081	DNMT3L	2.973142
RGS6	4.29006	Previous version conserved probe	2.959754
IL6A	4.189131		
SKIL	4.187362		
NA	4.095417		
SYVN1	4.048273		
Previous version conserved probe	4.01005		
APOD	4.006074		
SOCS4	3.99778		
SOCS2	3.975979		
TXNIP	3.974856		
STC2	3.92182		
Previous version conserved probe	3.905912		
XRN1	3.864944		
ARNT2	3.827677		
GKN2	3823828		
FOLR3	3.804816		
DNAJC27	3.797725		
CD14	3.782803		
ACSM1	3.779232		
TIPARP	3.768639		
TFPI2	3.75966		
IKZF5	3.737925		
VIMP	3.723051		
YOD1	3.722015		
CCL23	3.713859		
ZIC5	3.692965		
RCOR3	3.686028		
NKX3–2	3.684322		
HHAT	3.682812		
ARL5B	3.632136		
CBR4	3.631683		

(*Continued*)

Upregulated genes (*n* = 167)		Downregulated genes (*n* = 63)	
**Gene_symbol**	**Times**	**Gene_symbol**	**Times**
ZNF91-CNTF	3.624791		
ZNF579	3.567502		
PPP1R15A	3.566831		
KRTAP10–11	3.560625		
BEFB1	3.526151		
BTG1	3.515382		
NA	3.497635		
DNAJB2	3.477544		
LIN7C	3.473314		
ZNF100	3.457458		
OTUD1	3.445979		
ADAMTS16	3.424203		
FAM22F	3.420947		
ZFP36L2	3.406391		
RGPD2	3.403125		
NRAS	3.402121		
ACE	3.396323		
USP36	3.396017		
KLLN	3.382249		
PLEKHF2	3.376264		
MT1X	3.375341		
DNAJB9	3.368374		
TEMD6	3.349737		
CLDN1	3.34402		
KRT34	3.344014		
ZNF780A	3.338213		
BIRC3	3.327507		
DYRK2	3.304364		
PRND	3.301632		
TANC2	3.300915		
ZNF483	3.292883		
NAT8L	3.284288		
FGFR1OP2	3.278565		
C7orf61	3.273194		

(*Continued*)

Upregulated genes (*n* = 167)		Downregulated genes (*n* = 63)	
**Gene_symbol**	**Times**	**Gene_symbol**	**Times**
SSTR4	3.269397		
NEK7	3.26409		
CHST11	3.240821		
CREBRF	3.223459		
RGS2	3.219536		
AVIL	3.215913		
PRTG	3.213472		
CITED2	3.210328		
RASAL3	3.200928		
DUSP5	3.186504		
Previous version conserved probe	3.178196		
ANKRD12	3.174043		
FGFR1OP2	3.161886		
SOCS6	3.145414		
TROVE2	3.1427		
Previous version conserved probe	3.133142		
PLB1	3.119883		
RMND5A	3.117397		
STK35	3.08311		
ABL2	3.08292		
FAHD2A	3.078636		
PDK4	3.073212		
COL8A2	3.073066		
USP31	3.071179		
TRIO	3.049309		
ATF3	3.047638		
ZFP36L1	3.047542		
STK4	3.046791		
EFNB2	3.037967		
MAP4K5	3.034629		
SLC30A1	3.034295		
LAMA4	3.031349		
NA	3.030964		

(*Continued*)

Upregulated genes (*n* = 167)		Downregulated genes (*n* = 63)	
**Gene_symbol**	**Times**	**Gene_symbol**	**Times**
YOD1	3.013577		
Previous version conserved probe	3.007218		
NCSTN	3.003911		
AGR3	2.993321		
Previous version conserved probe	2.989411		
Previous version conserved probe	2.987623		
